# Supplementary material for: The Practice Guidelines for Multidose Drug Dispensing Need Revision—An Investigation of Prescription Problems and Interventions
Source: Pharmacy (Basel). 2021 Jan 6;9(1):13. doi: 10.3390/pharmacy9010013 (PMC7838985; doi:10.3390/pharmacy9010013)
Supplement: Supplementary file 1 [file pharmacy-09-00013-s001.pdf]

Date………….…… ☐Technician sign………….. ☐ Pharmacist sign……………

REGISTRATION OF INTERVENTIONS ON MULTIDOSE PRESCRIPTIONS

1. BACKGROUND INFORMATION

Type of patient: ☐ Private ☐ Nursing home ☐ Home care service

RX Reception: ☐ NA ☐ Fax ☐ E-mail ☐ Telephone ☐ Electronic ☐ Other:……………..............

2a. ERRORS ON THE MDD ORDER

☐ Missing ☐ Missing shipping address ☐ Illegible ☐ Other:…………………………………………………

2b. FORMAL ERRORS ON PRESCRIPTION(LIST)

☐ Date ☐ Patient's date of birth/name ☐ Missing approval for compassionate use ☐ Unknown MD

☐ Missing ☐ Missing signature ☐ Other:……………………………………………………………………….

2c. ERRORS ON PRESCTIPTION ITEM ☐ **Drug-shortage**

Patient: Year of birth:……….. Sex:……….

Prescriber: ☐ MD Community ☐ MD other

Prescription type: ☐ Medication list ☐ Single prescription

Description of problem: ………………………………………………………….………………….………………………......……….....

……….

……….

Problem (include information on drug, dose and use):

☐ Drug or strength ………….…………………………………………………………………………………………………….

☐ Dose/schedule ………….…………………………………………………………………………………………………….

☐ Dosage form …….

☐ Quantity/duration ☐ Drug-drug interactions ☐ Reimbursement ☐ CAVE

☐ Other: ………

3. INTERVENTION

☐ Contacted prescriber ☐ Contacted nursing home ☐ Contacted home care service

☐ Pharmacist's own judgement ☐ Patient profile/prescription history reviewed

Prescriber informed retrospectively: ☐ Yes ☐ No

4. RESULT:

☐ Added, discontinued or clarified drug or strength ☐ Received necessary documentation

☐ Changed or clarified dose/schedule ☐ Dispensed as prescribed/information given

☐ Changed or clarified dosage form ☐ Drug not dispensed

☐ Changed of clarified quantity/duration ☐ Dispensed without correction/clarification

☐ Changed or clarified reimbursement information

☐ Other:……..

5. TOTAL TIME SPENT(ca): ……………………….. min
